# Preoperative anxiety in children: bridging the gap between perception and observation

**DOI:** 10.1016/j.bjane.2026.844734

**Published:** 2026-02-02

**Authors:** Barbara Monique Calsolari Oliveira, Daniela Cristina Ikeda, Paulo Cesar Koch Nogueira, Mila Torii Corrêa Leite

**Affiliations:** a Universidade Federal de São Paulo, São Paulo, SP, Brazil; b Universidade Federal de São Paulo, Escola Paulista de Medicina, Department of Anesthesiology, São Paulo, SP, Brazil; c Universidade Federal de São Paulo, Escola Paulista de Medicina, Department of Pediatrics, São Paulo, SP, Brazil; d Universidade Federal de São Paulo, Escola Paulista de Medicina, Pediatric Surgery Division of Department of Surgery, São Paulo, SP, Brazil

Dear Editor Liana Azi,

Preoperative anxiety is a frequent and clinically relevant emotional response, particularly among pediatric patients and their caregivers.^1^ Despite its high prevalence, preoperative anxiety often goes unnoticed or is inadequately quantified in clinical practice. Observable behavioral signs and subjective perceptions do not always align, leading to an underestimation of children’s emotional distress before surgery. Understanding this mismatch between what is seen and what is felt ‒ between *observation* and *perception* ‒ is crucial for improving perioperative care.

A cross-sectional study was carried out from August 2022 to October 2023 in the pediatric surgical ward of a tertiary university hospital in São Paulo, Brazil. The study included 50 children aged 4–17 years undergoing elective surgery. Anxiety was evaluated using two validated instruments: the Modified Yale Preoperative Anxiety Scale (mYPAS),^2^ to assess behavioral manifestations, and the Visual Analogue Scale (VAS),^3^^,^^4^ which captures the subjective perception of anxiety reported by children aged ≥ 7 years. Data collection was carried out through convenience sampling by the same researcher in the inpatient unit on the day preceding surgery, in the presence of caregivers, and prior to the administration of any anxiolytic medication.

A total of 50 patients were interviewed, of whom 32/50 (64%) were male. No eligible patient declined to participate in the study. Among the patients, 28/50 (56%) had undergone at least one previous surgical procedure. Over 45/50 (90%) of patients reported no history of previous surgical or anesthetic complications. ROC analysis was used to assess the ability of patient-reported VAS to discriminate high anxiety defined by mYPAS ≥ 30. The analysis included 36 children aged ≥ 7 years with complete data. AUC and its 95% Confidence Interval were calculated using the DeLong method, and the optimal cut-off was identified using the Youden index.

Regarding preoperative anxiety assessed by the VAS in 36 patients, 8/36 (22%; 95% CI 11%–38%) reported the maximum score (10/10), representing the extreme upper bound of the scale. Overall, VAS scores showed a median of 5.0 (IQR 2.75–9.0), with a mean of 5.3 (SD = 3.5), indicating moderate anxiety levels in the cohort.

According to mYPAS assessed in 50 patients, the lowest score recorded was 23.4, observed in 12 patients, while the highest score was 83.4, observed in 1 patient. Using the established cut-off score of 30 to indicate anxiety, 29/50 (58%; 95% CI 43%–72%) patients were classified as anxious. In 36 children, patient-reported VAS showed limited discriminative performance, with an AUC of 0.51 (95% CI 0.32–0.71). The optimal cut-off identified by the Youden index was VAS = 6, yielding a sensitivity of 47% and a specificity of 59% (Fig. 1). Given the AUC close to 0.5 and the wide confidence interval, the ROC results indicate no evidence of meaningful discrimination between VAS and mYPAS in this small sample and should be interpreted as exploratory.

**Figure 1:**
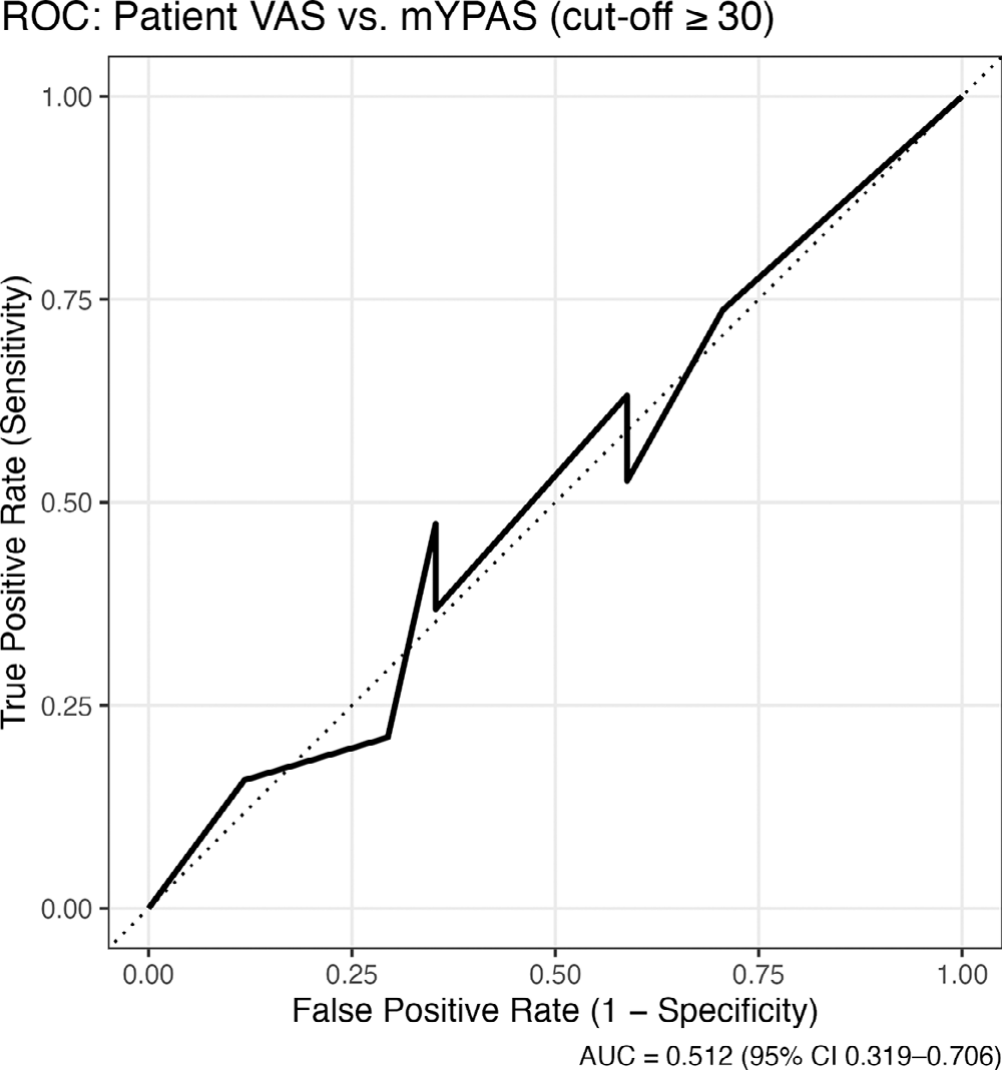
The discriminative validity of the patient’s self-reported anxiety (VAS 0–10) was tested using ROC analysis against the mYPAS cut-off (≥ 30). The area under the curve (AUC = 0.512; 95% CI 0.319–0.706) indicated no discriminative ability, suggesting that the self-perceived anxiety level did not correspond to the observational anxiety classification obtained with the mYPAS.

The main limitations of this study include the use of convenience sampling, the relatively small sample size, the heterogeneity of surgical procedures, and age-related limitations in the application of the VAS. The VAS lacks clearly defined anxiety cutoff points, and children’s responses may be influenced by parental presence and individual personality traits.

In conclusion, preoperative anxiety in pediatric surgical patients remains highly prevalent and multifaceted. In this exploratory study, discrepancies were identified between perceived and observed anxiety, indicating that neither instrument alone fully captures the construct.

## Conflicts of interest

The authors declare no conflicts of interest.
